# Global effects of forest modification on herpetofauna communities

**DOI:** 10.1111/cobi.13998

**Published:** 2022-11-21

**Authors:** Maider Iglesias‐Carrasco, Iliana Medina, Terry J. Ord

**Affiliations:** ^1^ Evolution and Ecology of Sexual Interactions Group Doñana Biological Station‐CSIC Sevilla Spain; ^2^ Research School of Biology Australian National University Canberra Australian Capital Territory Australia; ^3^ School of BioSciences University of Melbourne Melbourne Victoria 3010 Australia; ^4^ Evolution & Ecology Research Centre and the School of Biological, Earth and Environmental Sciences University of New South Wales Kensington New South Wales Australia

**Keywords:** anthropic habitats, fire, forestry, land conversion, logging, silviculture, conversión del suelo, incendios, hábitats antrópicos, silvicultura, tala, 火灾, 林业, 土地转换, 伐木, 造林

## Abstract

As the area covered by human‐modified environments grows, it is increasingly important to understand the responses of communities to the novel habitats created, especially for sensitive and threatened taxa. We aimed to improve understanding of the major evolutionary and ecological processes that shape the assemblage of amphibian and reptile communities to forest modifications. To this end, we compiled a global data set of amphibian and reptile surveys in natural, disturbed (burned, logged), and transformed (monocultures, polyspecific plantations) forest communities to assess the richness, phylogenetic diversity, and composition of those communities, as well as the morphological disparity among taxa between natural and modified forest habitats. Forest transformations led to a diversity reduction of 15.46% relative to the statistically nonsignificant effect of disturbances. Transformations also led to a community composition that was 39.4% dissimilar to that on natural forests, compared with 16.1% difference in disturbances. Modifications did not affect the morphological disparity of communities (*p* = 0.167 and 0.744), and we found little evidence of taxon‐specific responses to anthropic impacts. Monocultures and polyspecific plantations detrimentally affected the conservation and ecological value of both amphibian and reptile communities and altered the evolutionary processes shaping these communities, whereas forests with lower impact disturbances might, to some extent, serve as reservoirs of species. Although different mechanisms might buffer the collapse of herpetological communities, preserving remaining natural forests is necessary for conserving communities in the face of future anthropic pressures.

## Resumen

Con el aumento del área cubierta por entornos modificados por el humano, cada vez es más importante entender las respuestas que tienen las comunidades a los nuevos hábitats creados, especialmente en los taxones sensibles y amenazados. El objetivo de este estudio es mejorar el conocimiento sobre los principales procesos evolutivos y ecológicos que condicionan el ensamblado de las comunidades de anfibios y reptiles ante las modificaciones forestales. Con este fin, compilamos un conjunto de datos globales de los censos de anfibios y reptiles en las comunidades forestales naturales, perturbadas (taladas, incendiadas) y transformadas (monocultivos, plantaciones poliespecíficas) para valorar la riqueza, diversidad filogenética y composición de aquellas comunidades, así como la disparidad morfológica entre los taxones entre los hábitats forestales naturales y modificados. Las transformaciones forestales llevaron a una reducción de 15.46% de la diversidad en relación al efecto sin significancia estadística de las perturbaciones. Las transformaciones también derivaron en una composición comunitaria que fue 39.4% diferente a la de los bosques naturales, en comparación con el 16.1% de diferencia en las perturbaciones. Las modificaciones no afectaron la disparidad morfológica de las comunidades (*p* = 0.167 y 0.744) y no encontramos suficiente evidencia de respuestas específicas por taxón a los impactos antrópicos. Los monocultivos y las plantaciones poliespecíficas afectaron negativamente a la conservación y al valor ecológico de las comunidades de anfibios y reptiles y alteraron los procesos evolutivos que condicionan a estas comunidades, mientras que los bosques menos impactados podrían, hasta cierto punto, actuar como reservorios de especies. Sin embargo, mientras que diferentes mecanismos pueden amortiguar el colapso de las comunidades herpetológicas, se requiere la conservación de los bosques naturales para preservar las comunidades que se enfrentarán a presiones antrópicas.

## 摘要

随着人为改造环境的覆盖面积增加, 了解群落如何响应人类创造的新生境也越来越重要, 特别是对于敏感和受威胁的类群来说。本研究旨在进一步理解塑造两栖动物和爬行动物群落响应森林改造的主要演化及生态过程。为此, 我们编制了一个全球两栖动物和爬行动物调查数据集, 在自然、受干扰 (烧毁、砍伐) 和改造 (单一种植、多品种种植) 的森林中, 评估这些群落的丰富性、系统发育多样性和组成, 以及类群在自然和改造的森林生境之间的形态差异。结果表明, 森林改造导致生物多样性减少15.46%, 而干扰的影响在统计学上并不显著。改造后森林的群落组成与自然森林的群落组成有39.4%的差异, 而受干扰森林的差异为16.1%。此外, 森林改造并不影响群落的形态差异 (*p* = 0.167和0.744), 我们没有发现类群特异的对人为影响的响应。单一种植和多物种种植对两栖动物和爬行动物群落的保护和生态价值都产生了不利影响, 并改变了形成这些群落的演化过程, 而受干扰影响较小的森林在某种程度上可能成为物种的储藏库。然而, 虽然可能存在不同机制来缓冲两栖及爬行动物群落的崩溃, 但面对未来的人为压力, 保护剩余的自然森林对于保护群落仍十分必要。**【翻译: 胡怡思; 审校: 聂永刚】**

## INTRODUCTION

The area covered by human‐made modifications to forests is increasing globally, with a consequent rise in the number of endangered species (Tilman et al., 2017). In general, human‐driven modifications reduce local species diversity and change local community structures (Gibson et al., 2011). However, the response of species to human modifications varies across communities, types of modification, and taxonomic groups (Etard et al., 2021; Gibson et al., 2011; Newbold et al., 2015). Although some species disappear after forest modification, others successfully colonize and even thrive in the new habitat (Nowakowski et al., 2017; Thompson et al., 2016). Lack of understanding of which clades are more likely to perish or persist in different types of human‐modified areas or of how communities in disturbed areas are typically assembled (e.g., Gardner et al., 2007) hampers the effective design and implementation of conservation strategies.

Many anthropic landscapes are characterized by a simplified resource structure and a general loss of ecological niches, where colonizing or remaining taxa are often exposed to novel selective pressures (Tews et al., 2004). These characteristics can affect the biotic and abiotic processes by which local communities are assembled in human‐modified environments, for example, through impacts on competition, facilitation, predator–prey interactions, dispersal, environmental filtering, and the interaction of these factors (Cadotte & Tucker, 2017; Gerhold et al., 2015). The response of communities to these altered biotic and abiotic interactions are complex but are predicted to result in the loss of biodiversity, differences in species composition and abundances, alteration of intra‐ and interspecific interactions, and changes in functional traits (Mouillot et al., 2013).

To fully understand the evolutionary and ecological drivers of community responses to the anthropogenic disturbances of forests, it is essential to explore the ways in which community composition and structure are affected. The most obvious means to do this is by measuring species richness and diversity to provide estimates of taxonomic diversity and the abundance of each species an ecosystem can support. Measures of phylogenetic diversity can be used to quantify the level of phylogenetic relatedness of species in a community. This information can reveal a number of processes that operate at the community level. For example, low phylogenetic diversity can reflect niche conservatism or the tendency for closely related species to use similar ecological resources and subsequently occupy similar environments (Wiens & Graham, 2005). High phylogenetic diversity might occur when closely related and ecologically similar species are filtered from a community through competitive exclusion.

In the context of anthropogenically modified habitats, habitat modifications can result in the exclusion of species with particular morphological and functional traits (Todd et al., 2017). Yet, closely related species might still be able to coexist in a community following resource partitioning that reduces ecological competition. Such niche differentiation can in turn prompt evolutionary differentiation in functionally relevant morphology (character displacement) (Stuart & Losos, 2013). These outcomes on morphology can be captured through measures of morphological disparity (Webb et al., 2002). Taken together, assessing species diversity, phylogenetic diversity, and morphological disparity of a community can shed light on the complex mechanisms and processes that shape the community following habitat disturbance or transformation.

The extent, directionality, and type of community response to habitat modification are all likely to depend on the nature and intensity of the human modification and on the taxa affected. We evaluated the impact of some of the major forms of forest modifications on natural communities. We were specifically interested in exploring modifications that resulted in forest‐like systems, rather than the conversion of a forest into an environment vastly different in structure, such as urban areas or cleared agricultural land. In this context, we considered 2 general phenomena: disturbances to existing forests defined as those single or repeated catastrophic events that affect communities, such as logging or burning, and transformations of forests that reflect a major and permanent change to the environment, such as the replacement of natural forest with plantations.

We focused on amphibian and reptile communities due to their sensitivity to changes in habitat (e.g., Sinervo et al., 2010). Amphibians and reptiles are among the most threatened vertebrate groups in the world (IUCN, 2020). This likely reflects their increased vulnerability to land conversion because of their limited dispersal ability and microhabitat specialization. Amphibians and reptiles differ in a range of ecological traits, so different habitat modifications could affect amphibian and reptile communities differently. For instance, amphibians often show poor resistance to desiccation, have low thermal tolerance, and are associated with shady areas, so any habitat modification that leads to drier and hotter conditions is likely to have strong detrimental effects and affect species distribution (e.g., Watling & Braga, 2015). In contrast, reptiles are more heliothermic, so dry and hot conditions that are detrimental to amphibians could create favorable thermoregulatory conditions for many reptiles. Modification that leads to a closed canopy, which curtails basking and thermoregulation, would have negative consequences for lizard and snake communities. Given these ecological differences, we focused on these two animal groups in our exploration of the similarities and differences in their responses to a variety of human‐induced forest disturbances and transformations.

To learn about the evolutionary processes driving community composition in modified forests, we examined impacts of forest modification on species and phylogenetic diversity, community composition, and body size disparity. Body size can be easily and consistently measured for most species (Etard et al., 2020) and reflects a collective outcome of a host of ecologically relevant factors (e.g., diet, physiology, dispersal ability) (Roy et al., 2002; Tingley et al., 2010; White et al., 2007). In particular, changes in body size disparity within communities are expected to reflect the effects of environmental characteristics on many functional processes (e.g., Etienne & Olff, 2004) and on the evolutionary processes that have shaped the community in the modified habitat. To explore the effects of different forest modifications on herpetological communities, we compiled a global data set of published amphibian and reptile surveys across major disturbances (logged or burned) and modifications (agricultural monocultures and polyspecific cultures) of forested areas and compared the communities in modified forests with communities in nearby natural forest. We also evaluated the time elapsed since the last disturbance event as a means of testing the temporal recovery of resident communities.

We examined several hypotheses. First, forest transformations lead to decreased species and phylogenetic diversity compared with natural forests, whereas disturbances lead to either diversity increases or diversity decreases because many species (especially reptiles associated with open canopies) rely on such disturbances to create habitat (Viljur et al., 2022). Second, communities in transformed forests differ more from natural forests than communities in disturbed forests. In disturbed forests, communities become more diverse and more similar to the natural habitat as time since disturbance increases. Third, body size disparity differs between natural forests and modified habitats and is higher or lower depending on the type of species that remain in the community after modification. Fourth, anurans and reptiles react differently to forest disturbance and transformation. In general, anurans are most affected by disturbances that create drier and hotter conditions, whereas lizards and snakes are adversely affected by transformations that create closed and dark microclimatic conditions.

The effects of human‐made habitat modifications on specific community variables (e.g., species richness—Cordier et al., 2021) and taxonomic groups (e.g., reptiles only—Doherty et al., 2020) have been explored, but to our knowledge, we are the first to examine the ecological and evolutionary processes that shape both amphibian and reptile communities affected by different types of forest modification (based on the combination of species and phylogenetic diversity and morphological disparity). Our broad goal was to improve understanding of how human‐induced modification of forests affects the ecological and evolutionary distinctiveness of taxonomic communities that are especially vulnerable to environmental change.

## METHODS

### Data collection

To evaluate how the modification of forests affects amphibian and reptile communities, we compiled information on species presence and absence and abundance when present in monospecific and polyspecific plantations and in forest patches that have been logged or burned. In all cases, data were compared with amphibian and reptile surveys conducted in nearby natural forest in the same study as a surrogate for probable predisturbance community composition. Our aim was to explore the effects of modifications to forests that after modification still resembled a forest (e.g., with shrubs and trees). Therefore, we did not explore other human‐made transformations that lead to more profound changes in habitat characteristics, such as urban areas or agriculture lands.

We searched Google Scholar and the Web of Science with a combination of the following terms: (herpet*, *amphibian*, *reptile*, *lizard*, *frog*, *snake*) AND (*monoculture*, *plantation*, *anthropic forest*) OR (manage*, *fire*, burn*, *logging*). We supplemented this search with checks of cited literature in recent papers to add articles that might have been missed. We included any study that met the following criteria.

Included studies had to have surveyed amphibian or reptile or both communities in natural forests and any combination of monoculture or polyspecific plantations or burned or logged patches.

We relied on author descriptions to assign sites to habitat types: natural forests, monocultures, polyspecific plantations, or disturbed natural forests (burned or logged). Natural forests were largely undisturbed forest areas near or adjacent to the transformed or disturbed areas or were the same land‐cover type prior to disturbance. Most studies we included used these forests as explicit controls. We considered forests to be any area composed of trees, but the openness of the forest could differ (from closed‐canopy tropical forests to more open mallee forests). Monospecific plantations had a single tree or shrub species and consequently had very simplified structural environments. In many cases, the tree or shrub species planted were not native to the region. Polyspecific plantations had ≥2 tree or shrub species and consequently were structurally more complex than monocultures. Disturbed natural forests were forest patches exposed to fires, thinning, or logging activities. Although fires are a natural disturbance in some regions, intensive fires are likely to increase in the coming years due to climate change (e.g., Jolly et al., 2015), so understanding how communities respond to fire is important in predicting how species will be affected. Most of the areas in this category had been burned or logged within the last 30 years (2 exceptions: Ofori‐Boateng et al., 2013, one sampling site logged 33 years ago, and Mitchell et al. [1997], 1 sampling site logged 90 years ago). We recorded time since disturbance. We were specifically interested in logging and fire disturbances in forests, so we did not include studies of clearing for livestock farming or secondary forests that had been subject to other disturbances, such as cattle grazing. We only included studies in which taxa were clearly identified to species level. The raw data used in the manuscript have been uploaded to DRYAD (https://datadryad.org/stash/share/po6fxcOmSYQLUaOk0eTlSvoEFQ9x‐UkJ4kMX3d26S54).

Most studies sampled several patches of the same type of forest but presented data as a single estimate of species presence or absence and abundances for each forest type. To retain consistency with those studies, the few cases where the data were given separately for each site surveyed were similarly pooled to obtain a single estimate of the community for that given habitat type, and subsequent statistical analyses were based on within‐study comparisons. In the case of monospecific plantations, when a study surveyed areas composed of different species (e.g., eucalypt and pine monocultures), we considered these surveys of separated communities and treated them as such in the statistical analyses. Because our aim was to compare the entirety of an amphibian or reptile community, when a study sampled a location in more than one season (e.g., dry and wet seasons), we combined the survey outcomes. In the only case (e.g., Beirne et al., 2013), where the results for different sampling methods was provided (e.g., pitfall vs. acoustic detection), we used data from the method that detected more species in the less species‐rich area. We considered this a conservative approach because it favors estimates of greater diversity in the more depauperate area, resulting in higher similarity in estimates between forest types.

Body size data were based on the maximum adult body size estimate for a species reported by AmphibiaWeb (2019), Meiri (2008, 2018), and Oliveira et al. (2017). Body size for amphibians and reptiles that are not snakes was recorded as snout–vent length. In snakes, it was recorded as total length. We excluded data on urodeles, caecilians, and turtles and studies focused exclusively on salamanders because of low representation (e.g., most cases <2 species), which hampered calculation of body size disparity for some communities. Body size was not available for all species (53 amphibian and 1 lizard species), so we did not include these species in the analyses for body size disparity. We used snakes and lizards as categories, rather than focusing on functional categorization (e.g., pooling snakes with legless lizards), because snakes and lizards differ in important aspects of their biology and to ensure that all the categories used covered all possible microhabitats (i.e., from fossorial to arboreal).

Because the effect of human modifications on species communities and the time of recovery after disturbance can vary depending on climatic region (Newbold et al., 2020), we noted the biome of each study site (based on the 14 categories of Olson et al. [2001]). We simplified the original classification to four broad categories based on the ecoregions covered by our collection of studies: tropical forests, temperate forests, Mediterranean forest and shrubland, and savanna.

### Statistical analyses

All statistical analyses were conducted using R 3.5.1 (R Development Core Team).

To explore how forest disturbances and modifications shape amphibian and reptile responses, we computed for each community: species diversity (calculated as Simpson diversity index), phylogenetic diversity (PD), body size disparity, coefficient of variation in body size, and Jaccard and Simpson indices as proxies for compositional similarity and species turnover. Diversity and body size indices were calculated for each taxonomic group and each of the included modified and natural locations. Community similarity and dissimilarity indices (Jaccard and Simpson) were calculated for each taxonomic group in each of the modified forests and compared with the corresponding natural habitat from the same study. To control for differences in sampling method and effort between studies, study identity was added as a random effect in all models (see below) (details on calculations of each score in Appendix S1).

### Effects of habitat transformation and disturbance on communities

We used lmerTest and lme4 to implement separate random‐effects linear mixed models for each score of diversity, body size disparity, and community similarity or turnover to explore whether forest modifications affected these variables. Our models tested whether the response to each type of habitat modification varied among anurans, lizards, and snake communities; whether the response to each type of habitat modification varied among biomes; and when the taxa and the biome did not moderate community responses, whether there was a general effect of habitat type on community scores independent of taxa and biome. We used the same basic structure of predictors and followed the same choice of model selection for each score. First, we ran a full model that included interaction terms between the type of habitat (natural, disturbance, or transformation) and taxa (lizards, snakes, and anurans) and the type of habitat and biome (temperate, tropical, savanna, Mediterranean). Study identity was included as a random effect to account for variation in the data that arose due to studies differing in sampling methods or effort. When interactions were not statistically significant, we reapplied a reduced model without the interactions to provide a better parameterization of the main effects as a means of exploring the general effects of habitat type on the community scores (Appendix S2). We also compared the level of support for the reduced versus full (with interactions) models based on a second‐order correction of the Akaike information criterion (AICc). The model computed as having the lower value was interpreted as better supported. In all cases, where interaction terms were not statistically significant, the reduced model was consistently the better supported model.

To further explore the effects of modification type, we examined individual effects of the five separated habitat categories (natural, monoculture, polyspecific plantation, logged or burned) in instances where the main effect of forest type (i.e., in the reduced model) was statistically distinguishable from zero (see Appendix S2 for the representation of the steps followed during analysis). Due to the low representation of snakes in polyspecific and monoculture plantations, models considering each of the 5 habitats were repeated with and without snakes to check for any potential bias.

Slight modifications to the general model structure were made in the case of size disparity and phylogenetic diversity. When size disparity was the response variable, we also included phylogenetic diversity as predictor, given that phylogenetically diverse communities may exhibit higher size disparity. Because PD is often correlated with species richness, we included log_10_‐transformed community species richness as an offset term to assess whether the type of habitat affects the community phylogenetic diversity per species. This offset term therefore corrects our response variable to show whether a community had higher or lower PD per species and led to results identical to dividing PD by number of species in a community.

### Effects of time since disturbance on communities

In the case of logging and fires, we also hypothesized that the time elapsed since the last disturbance event affects communities and that their recovery capacity varies between taxa and biomes (e.g., Viljur et al., 2022). To explore this idea, for each community score we implemented a full model that included the interactions between the log_10_‐transformed time since the last disturbance event (in years) and taxa and between time and biome. To include natural forests in these analyses, we arbitrarily set these areas with an age of 100 years (unless otherwise specified in the original study). Again, study identity was added as a random effect to control for potential differences in study methodology and survey effort. We performed a global analysis without separating the type of disturbance to explore the generality of recovery time on community composition. When interactions were not statistically significant, we reran the reduced models without interactions to explore the general effect of time on community responses.

For all models, we transformed our data by using either log_10_ (in the case of *β*_sim_, PD) or the function PowerTransform (in the case of Simpson diversity, Jaccard, size disparity, and coefficient of variation of size) to increase the model fit and to better meet the assumptions of normality and heteroscedasticity of the residuals. When outliers were observed in plots, we repeated the models with these removed. Results excluding outliers were qualitatively unchanged, therefore we report results including all data points. We tested the statistical effect of model terms with the Anova function of the car package and the summary function in lme4. We reported the estimates of the Anova for general patterns (e.g., general effect of habitat type) and for models including interactions and used the estimates of the summary for specific comparisons within the main effect models (e.g., burned vs. natural forest comparison).

## RESULTS

**FIGURE 1:**
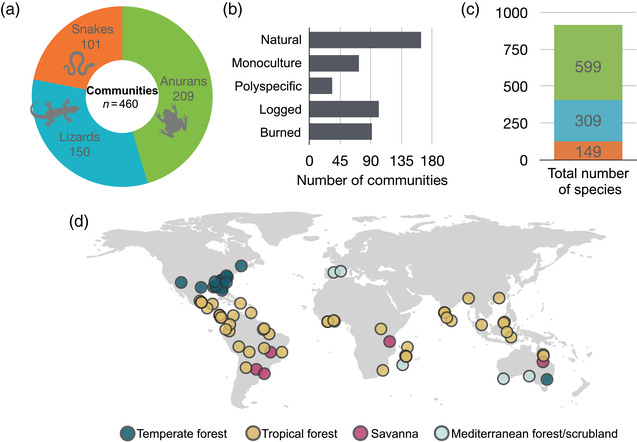
In surveys of 460 amphibian and reptilian communities, the (a) number of species surveyed by broad taxonomic groups, (b) forest types surveyed, (c) number of species surveyed, and (d) geographic location of forests (*n* = 76) in surveyed biomes

Our data set consisted of 460 communities that contained 309 lizard, 149 snake, and 599 amphibian species (Figure 1; Appendix S5). We focused analyses on communities with ≥2 species per taxonomic group (430 communities).

### Effects of habitat transformation and disturbance on communities

**FIGURE 2:**
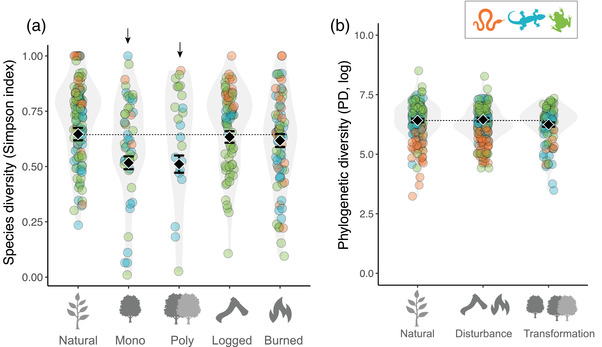
Effect of forest transformation (monoculture vs. polyspecific plantations) and disturbance (logged vs. burned) on (a) species diversity (Simpson index) and (b) phylogenetic diversity (values below dotted line, diversities lower in modified habitats than in natural forest; green, anurans; blue, lizards; orange, snakes; arrow, directionality of significant statistical effect). In panel (a), the 5 habitat types are shown separately because species diversity in transformations was statistically different from that in natural forests, whereas in panel (b) the 5 habitats are combined in broad types of modifications because none significantly affected phylogenetic diversity relative to that computed for natural forests.

We did not find any significant interaction between forest modification and taxa or biome for any of the variables examined (all *p* > 0.05) (summary of results in Appendix S4). Refitting models without interaction terms showed that transformed forests had lower species diversity (transformation vs. natural comparison: estimate [SE] = −0.098 [0.018], *t* = −5.376, *p* < 0.001) (Figure 2a) than natural and disturbed forests, but this was not the case for phylogenetic diversity per species (*p* = 0.458) (Figure 2b). When we explored the effects of each type of forest modification further, both polyspecific plantations and monocultures had lower species diversity than natural forests as did burned and logged forests (both with and without snakes) (all *p* < 0.038) (Figure 2a).

**FIGURE 3:**
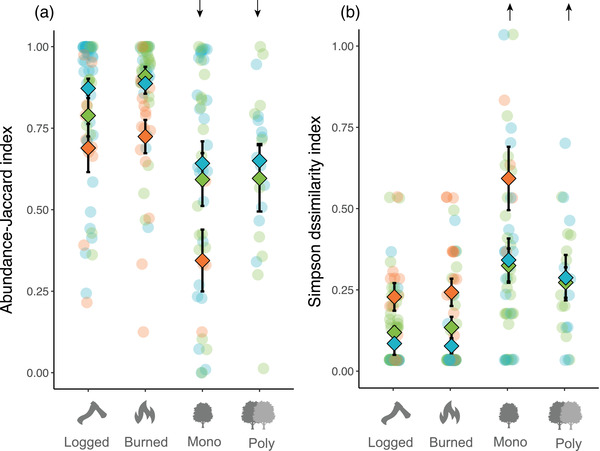
Effect of forest transformation (monoculture vs. polyspecific plantations) and disturbance (logged vs. burned) on community composition (a) Jaccard index and (b) Simpson index (green, anurans; blue, lizards; orange, snakes; arrow, directionality of significant statistical effects; reference condition, natural forest). Results are from a model with the 4 categories of modification (monoculture, polyspecific plantation, burned, and logged) separated because the different disturbances and transformations had statistically significant effects on both measures of community composition.

The difference in community composition between transformed and natural forests was greater than that between disturbed and natural forests (transformation vs. disturbance comparison: Jaccard index, estimate [SE] = −0.135 [0.0512], *t* = −2.641, *p* = 0.01; *β*_sim_, estimate = 0.453 [0.157], *t* = 2.876, *p* = 0.005) (Figure 3a,b). Communities were more dissimilar (*β*_sim_) from natural forests in monocultures than in logged forests (estimate = 0.554 [0.175], *t* = 3.171, *p* = 0.002) (similar result without snakes), but none of the other comparisons could be statistically distinguished from zero effect. In addition, we found a significant main effect for taxonomic group (*χ* ^2^ = 27.521, df = 2, *p* < 0.001). Snake communities in modified habitats were, in general, more dissimilar to communities in natural forest than anurans (*p* < 0.001), and lizards were more similar to communities in natural forest than those of anurans (*p* = 0.028). With the Jaccard index, communities were more dissimilar from natural forests in both monocultures (estimate = −0.139 [0.059], *t* = −2.67, *p* = 0.021) and polyspecific plantations (estimate = −0.157 [0.065], *t* = −2.406, *p* = 0.019) than in logged forests, but not than in burned forests (both *p* > 0.076) (Figure 3a). Models without snakes also showed that monocultures (*p* = 0.004), but not polyspecific plantations (*p* = 0.689), differed in dissimilarity from logged forests. We considered this last result in our interpretation of findings because it is more conservative. We found a general response of taxonomic group (*χ* ^2^ = 43.666, df = 2, *p* < 0.001) in which snake communities in modified habitats were more dissimilar to those in natural forests than both anuran and lizard communities (both *p* < 0.001).

**FIGURE 4:**
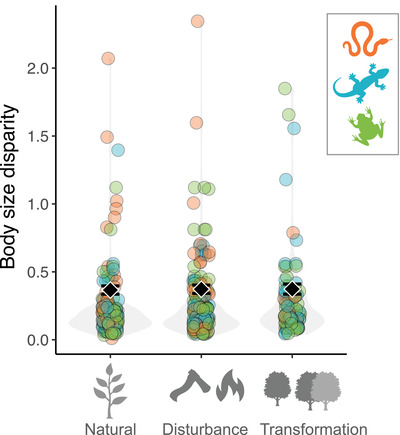
Effect of forest modification on body size of anurans (green), lizards (blue), and snakes (orange) in 3 habitat types (natural forests, transformations, and disturbances). Habitats are combined because separate categories of disturbance and transformation did not have statistically significant effects on community body size disparity.

The type of forest modification had no effect on body size disparity (*χ* ^2^ = 3.577, df = 2, *p* = 0.167) (Figure 4) or coefficient of variation of body size (*χ* ^2^ = 0.593, df = 2, *p* = 0.744).

### Effects of the time since disturbance on communities

**FIGURE 5:**
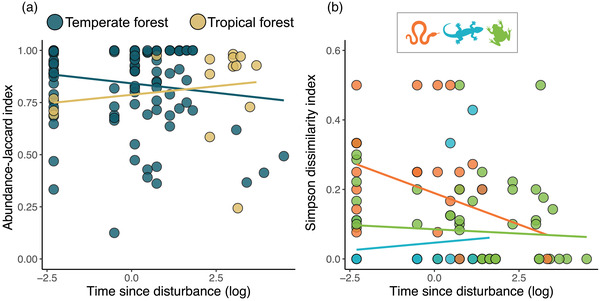
Effect of time since last disturbance on community composition (a) in tropical and temperate forests (as reflected in Jaccard similarity index) and (b) for each taxonomic group (based on Simpson dissimilarity index) (lines, direction of the effect)

We found a statistically significant interaction between the time elapsed since disturbance and the type of biome occupied by communities for the Jaccard index (*χ* ^2^ = 5.963, df = 1, *p* = 0.014) (Figure 5a). Specifically, disturbed forest communites in the tropics progressively increased in similarity to a predisturbed state over time, whereas temperate forests tended to decrease even further in similarity from a predisturbed state with time (although the slope was not significant). We also found a statistically significant interaction bewteen time and taxa for the score of *β*_sim_ (*χ* ^2^ = 6.889, df = 2, *p* = 0.032) (Figure 5b). That is, snake communities showed generally greater dissimilarities in disturbed patches than anurans and lizards (Figure 3b), but snakes had a strong recovery capacity and became similar to predisturbed natural forests over time. In contrast, anurans and lizards were in general less affected by the disturbance of the habitat and both groups remained at largely constant similarity levels over time. Interaction between time and taxa and time and biome and the main effect of time alone in the simplified models had no statistical effect on species or phylogenetic diversity per species or any measure of community size disparity (all *p* > 0.199) (Appendix S3).

## DISCUSSION

Our synthesis of global amphibian and reptile surveys showed that forest modifications resulted in a considerable loss of species diversity and other important changes in community composition. The response of ecological communities to human‐induced modifications of forested habitats depended, however, on the extent of the modification, and logging and burning disturbances seemed to have higher conservation value than transformations resulting in plantations. This finding is in accordance with previous studies that show disturbed forests can sustain similar species richness to natural forests (Gibson et al., 2011; Putz et al., 2012). In contrast, we found transformation of forests into plantations not only reduced diversity, but also resulted in a high degree of community changes and species turnover (captured by *β*_sim_). This is generally consistent with results of other studies that show different types of habitat modification often purge specialist species and encourage subsequent colonization by generalists (Evans et al., 2011; Newbold et al., 2018; Santos et al., 2014). More broadly, our results suggest that regenerating disturbed forests might, in some cases, partially mitigate the loss of natural forests, whereas the transformation of areas into plantations produces long‐term and detrimental effects.

The observed loss of diversity and the changes in community composition in forests transformed into plantations could be the result of a variety of changes in the forests. For example, plantations can lead to the structural simplification of the environment, the results of which are contraction of available niches, altered microclimate (e.g., more ground shade), and changes in the chemical environment due to the release of exotic leachates (Iglesias‐Carrasco, Wong, et al., 2022). All these alterations can affect amphibian and lizard behavior and physiology (Iglesias‐Carrasco, Cabido, et al., 2022) and lead to losses in species and functional diversity (Tews et al., 2004), which might in turn cascade into effects that prompt the ultimate collapse of the community (Dakos & Bascompte, 2014; Dunne & Williams, 2009).

Our results showed that despite diversity loss, modified forests were not linked to the loss of particular groups with specific body sizes, but instead maintained similar size disparities to those found in natural forests. One possibility is that certain species were initially lost following habitat simplification but then these were ultimately replaced by other colonizing species of comparable body size, although these invading species would presumably differ in other key life‐history traits and niche exploitation suited to living in plantations (Todd et al., 2017). This might be the case for snake communities, which showed a strong turnover in the species composition in modified habitats (based on *β*_sim_), but did not differ in their body size disparity. Alternatively, or in addition, communities in natural forests might support many ecologically and functionally redundant species that buffer these communities against the impacts of habitat simplification (Laliberté et al., 2010), allowing the maintenance of similar variation in morphological traits. If this is the case, our findings imply that communities subject to severe disruptions might avoid collapse because of a high degree of body size‐related functional redundancy in community composition in the original habitat.

We found no differences in body size disparity and phylogenetic diversity per species between natural and modified forests. This suggests that there were no strong effects of body size and phylogenetic relatedness on the ability of species to colonize these modified habitats. Our findings contrast with previous studies showing that the phylogenetic history of species can affect colonization success of modified habitats, manifesting in the loss of whole evolutionary distinct clades in such environments (Greenberg et al., 2018; Nowakowski et al., 2018). The fact that body size disparity did not show any pattern refutes the potential of ecological sorting based on competitive exclusion of morphologically similar species, or evolutionary character displacement, as the main evolutionary processes shaping communities in modified forests. This is because ecological sorting is likely to result in communities of species with higher phylogenetic and size disparity than expected by chance, whereas character displacement would result in communities having lower phylogenetic but higher size disparity than a random assemblage. Our results also indicated that the loss of species in transformed forests was not associated with environmental filters on body size, which is consistent with some previous studies (Doherty et al., 2020; Nowakowski et al., 2017), but contrasts with others that show size‐specific species losses associated with human‐modified habitats (Iglesias‐Carrasco et al., 2019; Nichols et al., 2007).

It is generally expected that communities in disturbed areas will recover with time. A disturbance event, such as a bushfire or logging, can induce important alterations in habitat structure (Bowman et al., 2009; Hu et al., 2013), such as the simplification of the canopy, which often leads to the creation of open environments with increased solar radiation and shrub growth. This new environment might benefit animals adapted to such conditions (e.g., Santos & Poquet, 2010) and displace forest‐dependent species. However, over time, succession often ends in a structurally similar environment to that before the disturbance, and as their habitat requirements are met, animal communities are expected to return in response to changes in vegetation.

Although some studies show support for the recovery capacity of communities (Dunn, 2004; Thompson & Donnelly, 2018), others do not (Cordier et al., 2021; Georgiev et al., 2020). Our results were mixed. We found evidence for the recovery capacity of communities for only some of the indices. Some communities in disturbed habitats began to resemble natural communities over time, and this recovery capacity seemed stronger in the tropics than for temperate forests (based on Jaccard results). We interpret this result with caution due to the small number of tropical locations with recent disturbances (Figure 5a), but it suggests that in tropical locations any disturbance of the forest has considerable short‐term effects on community composition. Such change in community composition might be the consequence of tropical forests being rapidly colonized by disturbance‐tolerant or terrestrial species immediately following a disturbance event, which are then pushed out in later stages as old‐growth forest specialists or arboreal species return with the progressive recovery of canopy height. In contrast, species in temperate forests are likely to historically occupy a simpler forest structure with a long history of human management, so disturbances might impose less pressure on these communities than in tropical biomes. Nevertheless, there was a general lack of any change over time for most of the variables examined. One potential explanation is that amphibian and reptile communities seem to be quite resilient to forest disturbances (e.g., similar PD or size disparity in disturbed and natural forests) and to not require recovery of the community to its original state. Alternatively, it is possible that for some of the measured indices the time elapsed was not adequate to detect community recovery. Most included studies surveyed communities relatively soon after the disturbance (e.g., <5 years). Some communities recover only after many decades (e.g., up to 100 years in some habitats [Nimmo et al., 2012]). In other communities, species richness remains similar in disturbed and undisturbed habitats for some years after disturbance; changes in species richness are detected only later (Viljur et al., 2022).

Anuran, lizard, and snake community composition responded differently to habitat modifications. In general, snake communities in modified habitats were more dissimilar from natural forests than anurans and lizards. This suggests that snakes experienced a higher turnover of species when natural forests were disturbed or replaced with plantations. However, we also found that snake communities had a strong recovery capacity and that species composition and abundances progressively became similar to those of the original natural forest. The high turnover of snakes might be the result of specialist top‐predator carnivore species abandoning the transformed forests (Todd et al., 2017) as species from lower trophic positions colonize the novel habitat. This would be consistent with studies showing that carnivore species are often more strongly affected by anthropic habitats than species from other feeding guilds (Keinath et al., 2017). However, we cannot discard the possibility that the high turnover detected reflects the difficulty of detecting snakes in the wild and the consequent potential randomness in the species and abundances detected during the samplings.

Our results also show that lizards seem to be more resilient to habitat disturbances than anurans (based on *β*_sim_), which might reflect a higher tolerance of lizard species to the potential drier conditions created after disturbance. Beyond that, amphibians and reptiles seemed to respond in a similar way to habitat modifications; we did not find any other moderating effect of taxonomic group. It might be expected that amphibians are the more likely group to exhibit detrimental effects after forest disturbances because of the often‐drastic reduction in water sources for breeding in human‐affected environments (Koralay & Kara, 2018). Amphibians generally have poor resistance to desiccation and low thermal tolerance that would presumably result in amphibians being less likely to remain in, or colonize, the often drier and hotter environment created by habitat disturbances (Bowman et al., 2009; Gardner et al., 2007; Hu et al., 2013). In contrast, reptiles might be expected to respond more strongly to the replacement of natural forests by plantations, which are often characterized by closed canopies and increased shade that impedes thermoregulation. Yet based on the community indices we measured, it seems amphibians and reptiles respond in a similar way to the altered conditions in modified forests (e.g., changes in shade availability, leaf litter characteristics, and presence of coarse woody debris and simplified forest structure) and that severe habitat transformations will pose an acute challenge for both amphibian and reptile conservation in an increasingly altered world.

As the area covered by human‐modified forests continues to increase around the world, it is critical to understand how communities respond to this—often drastic—environmental change. We found that anthropic modifications to forests can threaten herpetological communities and that some specific modifications, such as the transformation of natural forests to plantations, are more detrimental than disturbances from burning and especially logging. However, our results in combination with those of other studies (Dunn, 2004; Thompson & Donnelly, 2018) suggest that the community dynamics of disturbed forests are complex, which might explain the discrepancies between various other studies on the recovery capacity of ecological communities during succession. Such conflicting results could reflect differences between studies, including the taxa and variables studied or the biotic and abiotic factors of the environments investigated, such as temperature, humidity, forest structure, competition, and predator–prey interactions (e.g., Díaz‐García et al., 2020; Herrera‐Montes & Brokaw, 2010). However, by identifying how communities respond to land modification at a broad, global level (as we have done here), one can begin to identify the evolutionary and ecological processes that shape such communities. By doing so, there is the possibility of designing plantations in a way that better promotes the coexistence of human activities and biodiversity. Indeed, the conservation of amphibians and reptiles in human‐affected areas will almost certainly depend on understanding the impacts of environmental change on reshaping the morphological and functional structure of these species communities. Our study is an important additional step toward that goal.

## Supporting information

Appendix
S1. Calculations of the variables used
S2. The decision path followed in statistical analyses
S3. Effect of time since disturbance event on a) phylogenetic diversity, b) body size disparity, and c) species diversity in anurans (green), lizards (blue), snakes (orange)
S4. Summary of the models applied following the steps outlined in Fig S1 and the general results obtained
S5. References used for extracting raw data: Click here for additional data file.
