# Supplementary material for: Global effects of forest modification on herpetofauna communities
Source: Conserv Biol. 2022 Nov 21;37(1):e13998. doi: 10.1111/cobi.13998 (PMC10099509; doi:10.1111/cobi.13998)
Supplement: Supplementary file 1 — Appendix S1. Calculations of the variables used S2. The decision path followed in statistical analyses S3. Effect of time since disturbance event on a) phylogenetic diversity, b) body size disparity, and c) species diversity in anurans (green), lizards (blue), snakes (orange) S4. Summary of the models applied following the steps outlined in Fig S1 and the general results obtained S5. References used for extracting raw data: [file COBI-37-0-s001.docx]

**Appendix**

**S1. Calculations of the variables used:**

Species and phylogenetic diversity: we first calculated the species diversity of a given community using the Simpson’s diversity index. For this index we only included the studies that provided clear abundance data of the species found (55 out of 76 studies). Second, we calculated the phylogenetic diversity of each community using the function PD in the package *picante* (Kembel et al. 2010). For this calculation we used recent phylogenetic estimates that included all species of squamates (Tonini et al. 2016) and amphibians (Jetz & Pyron 2018). We calculated the maximum clade credibility (MCC) tree as the summary of the posterior distribution of published trees. Using the MCC tree is comparable, in terms of controlling for branch and topology uncertainty, to sampling many times from the posterior distribution and estimating a mean PD for each community.

Body size disparity: We calculated the mean minimum size disparity for each community and taxa (snakes, lizards and anurans) as the average difference in log10-transformed body size between each species and the next species closest in size within each community. In addition, we calculated the coefficient of variation in body size of the communities by dividing the community body size standard deviation by the average in order to get further insight into the kind of processes that might have shaped the assemblages.

Community dissimilarity indices: We examined the compositional similarity of communities between the natural habitat and each modified habitat within each study using the abundance-based Jaccard’s index (Chao et al. 2005) and the Simpson’s dissimilarity index (β_sim_). Again, we only used those studies that reported abundance data. In the case of Jaccard, we measured the pairwise similarities between the natural and their corresponding transformed/disturbed habitat as the proportion of shared species abundances between both habitats. This index has been suggested to provide a good estimate of changes in species composition between anthropic and non-anthropic habitats (Chao et al. 2005). We used a 100% similarity benchmarked to the natural forest as a gross baseline for change in community composition. Ideally, the similarity of species among different sampling sites within natural forests would have provided a more realistic benchmark (as in e.g., Ewers et al. 2009), given that comparisons among replicate samples of the same habitat are unlikely to have perfect similarity, but these should nevertheless be more similar to one another than comparisons made across disparate habitat types. Unfortunately, data for multiple sampling of the natural habitat was almost always pooled into a single estimate (see Methods in main text), so this type of benchmarking was not possible. Regardless, our general focus was on the magnitude of dissimilarity *among* habitat modification categories, rather than absolute dissimilarity for a given habitat type *per se*.

Last we computed β_sim_, which measures community similarities with the effect of species richness removed, to provide a more explicit indication of species turnover in communities (Koleff et al. 2003). We calculated this score as the minimum unique species richness of a community divided by the sum of the minimum species richness and the number of interactions between the communities (Koleff et al. 2003). A score converging on a value of 0 represents low species turnover and subsequently high species similarity between communities (with 0 itself being complete species overlap).

**References**

Chao A, Chazdon RL, Shen TJ. 2005. A new statistical approach for assessing similarity of species composition with incidence and abundance data. Ecology Letters **8**:148–159.

Ewers RM, Kapos V, Coomes DA, Lafortezza R, Didham RK. 2009. Mapping community change in modified landscapes. Biological Conservation **142**:2872–2880.

Jetz W, Pyron RA. 2018. The interplay of past diversification and evolutionary isolation with present imperilment across the amphibian tree of life. Nature Ecology & Evolution **2**:850–858.

Kembel SW, Cowan PD, Helmus MR, Cornwell WK, Morlon H, Ackerly DD, Blomberg SP, Webb. CO. 2010. Picante: R tools for integrating phylogenies and ecology. Bioinformatics **26**:1463–1464.

Koleff P, Gaston KJ, Lennon JJ. 2003. Measuring beta diversity for presence-absence data. Journal of Animal Ecology **72**:367–382.

Tonini J, Beard KH, Barbosa R, Jetz W, Pyron RA. 2016. Fully-sampled phylogenies of squamates reveal evolutionary patterns in threat status. Biological Conservation **204**:23–31.

**S2.** The decision path followed in statistical analyses. The model structure was designed to address three general questions: Do anurans, lizards and snakes respond differently to the modifications of the habitat? Do herpetological communities in general respond differently to habitat modifications depending on the geographical region (biome)? Do communities recover as the time elapsed since the last disturbance increases? All models included Study ID as a random effect to control for potential differences in methodology and survey effort among studies.

**S3.** Effect of **time** since disturbance event on a) phylogenetic diversity, b) body size disparity, and c) species diversity in anurans (green), lizards (blue), snakes (orange). Lines connect all communities from the same study.

**
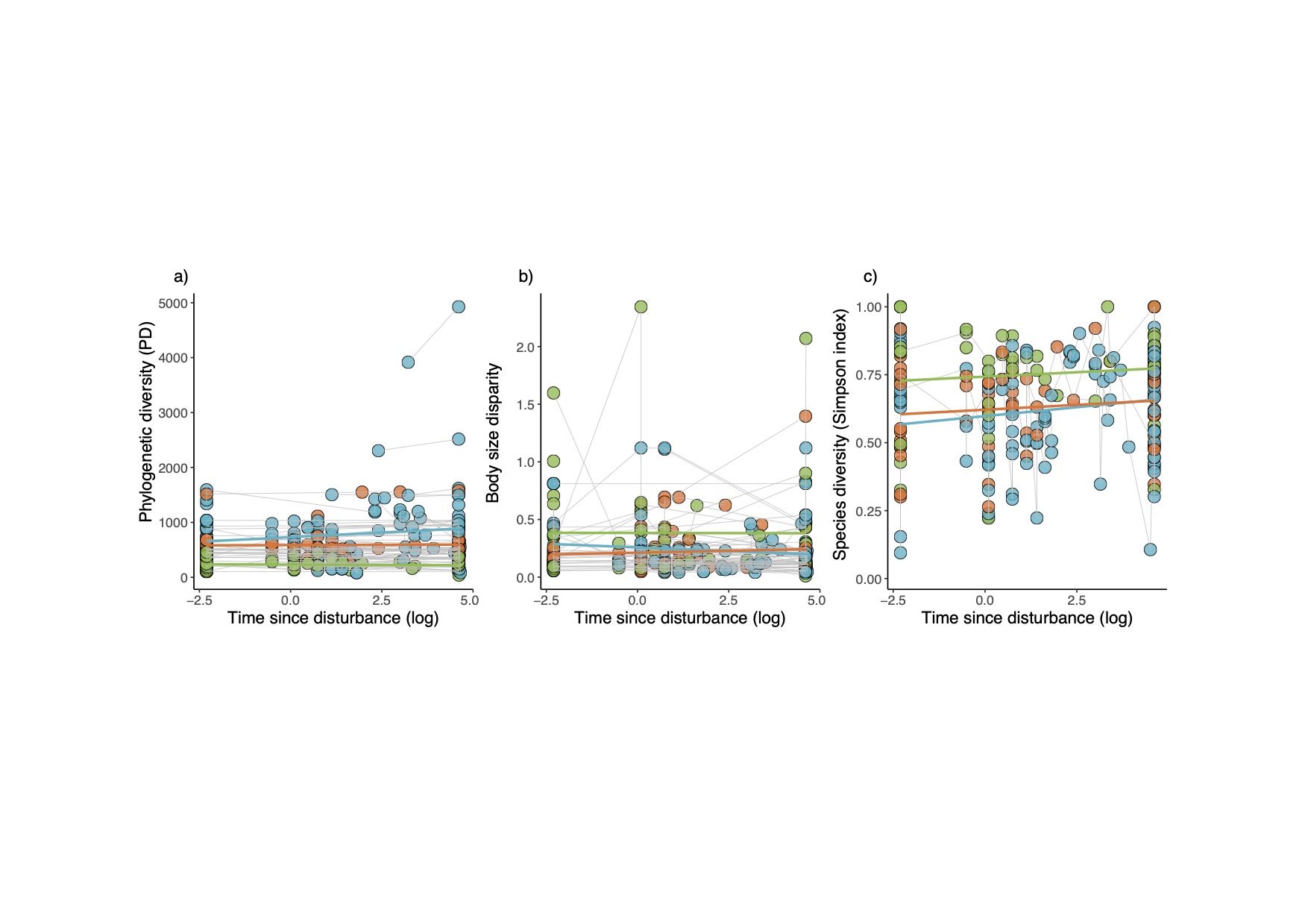
**

**S4**. Summary of the models applied following the steps outlined in Fig S1 and the general results obtained.

| **Score** | **Model structure** | **Result** | **Interpretation** |
| --- | --- | --- | --- |
|  | **General models to explore effects of the type of habitat modification** | | |
|  |  |  |  |
| Species diversity (Simpson) | Full model: simpson~Taxa*Habitat_type + Biome*Habitat_type + (1\|Study) | Interactions NS |  |
|  | Reduced model: simpson~Taxa + Habitat_type + Biome+ (1\|Study) | **Habitat_type significant** | Transformed forests lower diversity |
|  | Five-habitat model: simpson~Taxa + Habitat_complex + Biome + (1\|Study) | **Habitat_complex significant** | Monocultures and polycultures lower diversity than the rest |
|  |  |  |  |
| Phylogenetic diversity | Full model: PD~Taxa*Habitat_type + Biome*Habitat_type + offset(log(Sp_richness) + (1\|Study) | Interaction NS |  |
|  | Reduced model: PD~Taxa + Habitat_type + Biome + offset(log(Sp_richness) + (1\|Study) | Main effect of habitat_type NS | |
|  |  |  |  |
| Jaccard similarity index | Full model: jaccard~Taxa*Habitat_type + Biome*Habitat_type + (1\|Study) | Interactions NS |  |
|  | Reduced model: jaccard~Taxa + Habitat_type + Biome+ (1\|Study) | **Habitat_type significant** | Transformed habitats more dissimilar than disturbed habitats |
|  | Five-habitat model: jaccard~Taxa + Habitat_complex + Biome + (1\|Study) | **Habitat_complex significant** | Monocultures and polycultures more dissimilar than logged forests |
|  |  | **Taxa significant** | Snake communities more dissimilar than anurans |
|  |  |  |  |
| Simpson dissimilarity index | Full model: simpsondis~Taxa*Habitat_type + Biome*Habitat_type + (1\|Study) | Interaction NS |  |
|  | Reduced model: simpsondis~Taxa + Habitat_type + Biome+ (1\|Study) | **Habitat_type significant** | Transformed habitats more dissimilar than disturbed habitats |
|  | Five-habitat model: simpsondis~Taxa + Habitat_complex + Biome + (1\|Study) | **Habitat_complex significant** | Monocultures more dissimilar than logged forests |
|  |  | **Taxa significant** | Snake communities more dissimilar than anurans; lizards less dissimilar than anurans |
|  |  |  |  |
| Size disparity | Full model: size_disp~Taxa*Habitat_type + Biome*Habitat_type + PD + (1\|Study) | Interaction NS |  |
|  | Reduced model: size_disp~Taxa + Habitat_type + Biome + PD + (1\|Study) | Main effect of habitat_type NS | |
|  |  |  |  |
| Coefficient of variation of body size | Full model: coeff_size~Taxa*Habitat_type + Biome*Habitat_type + (1\|Study) | Interaction NS |  |
|  | Reduced model: coeff_size~Taxa + Habitat_type + Biome + (1\|Study) | Main effect of habitat_type NS | |
|  |  |  |  |
|  | **Models of time since last disturbance** |  |  |
|  |  |  |  |
| Species diversity (Simpson) | Full model: simpson~Taxa*log(time) + Biome*log(time) + (1\|Study) | Interactions NS |  |
|  | Reduced model: simpson~Taxa + log(time) + Biome+ (1\|Study) | Main effect of time NS |  |
|  |  |  |  |
| Phylogenetic diversity | Full model: PD~Taxa*log(time) + Biome*log(time) + offset(log(Sp_richness) + (1\|Study) | Interactions NS |  |
|  | Reduced model: PD~Taxa + log(time) + Biome + offset(log(Sp_richness) + (1\|Study) | Main effect of time NS |  |
|  |  |  |  |
| Jaccard similarity index | Full model: jaccard~Taxa*log(time) + Biome*log(time) + (1\|Study) | **Biome*time interaction significant** | Communities in the tropics recover better than in temperate areas |
|  |  |  |  |
| Simpson dissimilarity index | Full model: simpsondis~Taxa*log(time) + Biome*log(time) + (1\|Study) | **Taxa*time interaction significant** | Snake communities become more similar to original with time |
|  |  |  |  |
| Size disparity | Full model: size_disp~Taxa*log(time) + Biome*log(time) + PD + (1\|Study) | Interactions NS |  |
|  | Reduced model: size_disp~Taxa + log(time) + Biome + PD + (1\|Study) | Main effect of time NS |  |
|  |  |  |  |
| Coefficient of variation of body size | Full model: coeff_size~Taxa*log(time) + Biome*log(time) + (1\|Study) | Interactions NS |  |
|  | Reduced model: coeff_size~Taxa + log(time) + Biome + (1\|Study) | Main effect of time NS |  |

**S5. References used for extracting raw data:**

Adum GB, Eichhorn MP, Oduro W, Ofori-Boateng C, Rödel MO. 2013. Two-stage recovery of amphibian assemblages following selective logging of tropical forests. Conservation Biology **27**:354–363.

Amo L, López P, Martín J. 2007. Natural oak forest vs. ancient pine plantations: Lizard microhabitat use may explain the effects of ancient reforestations on distribution and conservation of Iberian lizards. Biodiversity and Conservation **16**:3409–3422.

Angarita O, Motes AC, Renjifo JM. 2015. Amphibians and reptiles of an agroforestry system in the Colombian Caribbean. Amphibian & Reptile Conservation **8**:19–38.

Beirne C, Burdekin O, Whitworth A. 2013. Herpetofaunal responses to anthropogenic habitat change within a small forest reserve in Eastern Ecuador. Herpetological Journal **23**:209–219.

Bennett S, Whitfield Gibbons J, Glanvillle J. 2017. Terrestrial activity, abundance and diversity of amphibians in differently managed forest types. The American Midland Naturalist **103**:412–416.

Brown GW. 2001. The influence of habitat disturbance on reptiles in a Box-Ironbark eucalypt forest of south-eastern Australia. Biodiversity and Conservation **10**:161–176.

Cano PD, Leynaud GC. 2010. Effects of fire and cattle grazing on amphibians and lizards in northeastern Argentina (Humid Chaco). European Journal of Wildlife Research **56**:411–420.

Correa FS, Juen L, Rodrigues LC, Silva-Filho HF, Santos-Costa MC. 2015. Effects of oil palm plantations on anuran diversity in the eastern Amazon. Animal Biology **65**:321–335.

Cortés-Gómez AM, Castro-Herrera F, Urbina-Cardona JN. 2013. Small changes in vegetation structure create great changes in amphibian ensembles in the Colombian Pacific rainforest. Tropical Conservation Science **6**:749–769.

Costa BM, Pantoja DL, Vianna MCM, Colli GR. 2013. Direct and short-term effects of fire on lizard assemblages from a neotropical savanna hotspot. Journal of Herpetology **47**:502–510.

Cruz-Elizalde R, Berriozabal-Islas C, Hernández-Salinas U, Martínez-Morales MA, Ramírez-Bautista A. 2013. Amphibian species richness and diversity in a modified tropical environment of central Mexico. Tropical Ecology **57**:407–417.

Cunningham SC, Babb RD, Jones TR, Taubert BD, Vega R. 2002. Reaction of lizard populations to a catastrophic wildfire in a central Arizona mountain range. Biological Conservation **107**:193–201.

D’Cruze N, Kumar S. 2011. Effects of anthropogenic activities on lizard communities in northern Madagascar. Animal Conservation **14**:542–552.

Da Cunha Bitar YO, Juen L, Pinheiro LC, Santos-Costa MC Dos. 2015. Anuran beta diversity in a mosaic anthropogenic landscape in transitional Amazon. Journal of Herpetology **49**:75–82.

Driscoll DA, Smith AL, Blight S, Maindonald J. 2012. Reptile responses to fire and the risk of post-disturbance sampling bias. Biodiversity and Conservation **21**:1607–1625.

Enge KM, Marion WR. 1986. Effects of clearcutting and site preparation on herpetofauna of a North Florida flatwoods. Forest Ecology and Management **14**:177–192.

Ernst R, Linsenmair KE, Rödel MO. 2006. Diversity erosion beyond the species level: Dramatic loss of functional diversity after selective logging in two tropical amphibian communities. Biological Conservation **133**:143–155.

Faria D, Paciencia MLB, Dixo M, Laps RR, Baumgarten J. 2007. Ferns, frogs, lizards, birds and bats in forest fragments and shade cacao plantations in two contrasting landscapes in the Atlantic forest, Brazil. Biodiversity and Conservation **16**:2335–2357.

Faruk A, Belabut D, Ahmad N, Knell RJ, Garner TWJ. 2013. Effects of oil-palm plantations on diversity of tropical anurans. Conservation Biology **27**:615–624.

Floyd TM, Russell KR, Moorman CE, Lear DH Van, Guynn DC, Lanham JD. 2002. Effects of prescribed fire on herpetofauna within hardwood forests of the Upper Piedmont of South Carolina: a preliminary analysis. Proceedings of the eleventh biennial southern silvicultural research conference:123–127.

Folt B, Reider KE. 2013. Leaf-litter herpetofaunal richness, abundance, and community assembly in mono-dominant plantations and primary forest of northeastern Costa Rica. Biodiversity and Conservation **22**:2057–2070.

Fredericksen NJ, Fredericksen TS. 2002. Terrestrial wildlife responses to logging and fire in a Bolivian tropical humid forest. Biodiversity and Conservation **11**:27–38.

Gallmetzer N, Schulze CH. 2015. Impact of oil palm agriculture on understory amphibians and reptiles: A Mesoamerican perspective. Global Ecology and Conservation **4**:95–109.

Gardner TA, Ribeiro-Júnior MA, Barlow J, Ávila-Pires TCS, Hoogmoed MS, Peres CA. 2007. The value of primary, secondary, and plantation forests for a neotropical herpetofauna. Conservation Biology **21**:775–787.

Germano JM, Sander JM, Henderson RW, Powell R. 2003. Herpetofaunal communities in Grenada: A comparison of altered sites, with an annotated checklist of Grenadian Amphibians and Reptiles. Caribbean Journal of Science **39**:68–76.

Gillespie G, Howard S, Lockie D, Scroggie M, Boeadi. 2005. Herpetofaunal richness and community structure of offshore islands of Sulawesi, Indonesia. Biotropica **37**:279–290.

Gillespie GR, Ahmad E, Elahan B, Evans A, Ancrenaz M, Goossens B, Scroggie MP. 2012. Conservation of amphibians in Borneo: Relative value of secondary tropical forest and non-forest habitats. Biological Conservation **152**:136–144.

Glor RE, Flecker AS, Benard MF, Power AG. 2001. Lizard diversity and agricultural disturbance in a Caribbean forest landscape. Biodiversity and Conservation **10**:711–723.

Greenberg CH. 2001. Response of reptile and amphibian communities to canopy gaps created by wind disturbance in the southern Appalachians. Forest Ecology and Management **148**:135–144.

Guerra C, Aráoz E. 2015. Amphibian diversity increases in an heterogeneous agricultural landscape. Acta Oecologica **69**:78–86.

Heinen JT. 1992. Comparisons of the leaf litter herpetofauna in abandoned cacao plantations and primary rain forest in Costa Rica: Some implications for faunal restoration. Biotropica **24**:431.

Hinde R, Corti G, Fanning E, Jenkins RKB. 2001. Anurans in the kilombero valley, tanzania: Comparison between miombo woodland, evergreen forest and teak plantations. Journal of the Herpetological Association of Africa **50**:35–39.

Hobbs R, Catling PC, Wombey JC, Clayton M, Atkins L, Reid A. 2003. Faunal use of bluegum (*Eucalyptus globulus*) plantations in southwestern Australia. Agroforestry Systems **58**:195–212.

Jenkins RKB, Brady LD, Bisoa M, Rabearivony J, Griffiths RA. 2003. Forest disturbance and river proximity influence chameleon abundance in Madagascar. Biological Conservation **109**:407–415.

Jongsma G, Hedley R, Duraes R, Karubian J. 2014. Amphibian diversity and species composition in relation to habitat type and alteration in the Mache-Chindul reserve, Northwest Ecuador. Herpetologica **70**:34–46.

Kanowski JJ, Reis TM, Catterall CP, Piper SD. 2006. Factors affecting the use of reforested sites by reptiles in cleared rainforest landscapes in tropical and subtropical Australia. Restoration Ecology **14**:67–76.

Keyser PD, Sausville DJ, Ford WM, Schwab DJ, Brose, Patrick H. 2004. Prescribed fire impacts to amphibians and reptiles in shelterwood-harvested oak-dominated forests. Virginia Journal of Science **55**:159–168.

Kilpatrick ES, Kubacz DB, Guynn DC, Lanham JD, Waldrop TA. 2004. The effects of prescribed burning and thinning on herpetofauna and small mammals in the Upper Piedmont of South Carolina: preliminary results of the National Fire and Fire Surrogate Study. Proceedings of the 12th biennial southern silvicultural research conference:18–22.

Kirkland G, Snoddy H, Amsler T. 1996. Impact of fire on small mammals and amphibians in a central Appalachian deciduous forest. The American Midland Naturalist **135**:253–260.

Konopik O, Steffan-dewenter I, Grafe TU. 2015. Effects of logging and oil palm expansion on stream frog communities on Borneo, Southeast Asia **47**:636–643.

Krishna SN, Krishna SB, Vijayalaxmi KK. 2005. Variation in anuran abundance along the streams of the Western Ghats, India. Herpetological Journal **15**:167–172.

Kurz DJ, Nowakowski AJ, Tingley MW, Donnelly MA, Wilcove DS. 2014. Forest-land use complementarity modifies community structure of a tropical herpetofauna. Biological Conservation **170**:246–255.

Langford GJ, Borden JA, Major CS, Nelson DH. 2007. Effects of prescribed fire on the herpetofauna of a Southern Mississippi pine savanna. Herpetological Conservation and Biology **2**:135–143.

Litt AR, Provencher L, Tanner GW, Franz R. 2001. Herpetofaunal responses to restoration treatments of longleaf pine sandhills in Florida. Restoration Ecology **9**:462–474.

Luja VH, Herrando-Pérez S, González-Solís D, Luiselli L. 2008. Secondary rain forests are not havens for reptile species in tropical Mexico. Biotropica **40**:747–757.

Machado I, Maltchik L. 2010. Can management practices in rice fields contribute to amphibian conservation in southern Brazilian wetlands ? Aquatic conservation **46**:39–46.

Macip-Ríos R, Muńoz-Alonso a. 2008. Lizard diversity in coffee crops and primary forest in the Soconusco Chiapaneco . Revista Mexicana de Biodiversidad **79**:185–195.

Matthews CE, Moorman CE, Greenberg CH, Waldrop TA. 2010. Response of reptiles and amphibians to repeated fuel reduction treatments. Journal of Wildlife Management **74**:1301–1310.

McLeod RF, Gates JE. 1998. Response of herpetofaunal communities to forest cutting and burning at Chesapeake Farms, Maryland. American Midland Naturalist **139**:164–177.

Mitchell JC, Pagels JF, Buhlmann KA, Mitchell JC, Rinehart SC, Pagels JF, Buhlmann KA. 1997. Factors influencing amphibian and small mammal assemblages in central Appalachian forests. Forest Ecology and Management **96**:65–76.

Moseley KR, Castleberry SB, Schweitzer SH. 2003. Effects of prescribed fire on herpetofauna in bottomland hardwood forests. Southeastern Naturalist **2**:475–486.

Mott B, Alford RA, Schwarzkopf L. 2010. Tropical reptiles in pine forests: Assemblage responses to plantations and plantation management by burning. Forest Ecology and Management **259**:916–925.

Murrieta-galindo R, López-barrera F, González-romero A, Parra-Olea G. 2013. Matrix and habitat quality in a montane cloud-forest landscape: amphibians in coffee plantations in central Veracruz , Mexico. Wildlife Research **40**:25–35.

Mushinsky H. 1985. Fire and the Florida Sandhill herpetofaunal community : with special attention to responses of Cnemidophorus sexlineatus. Herpetologica **41**:333–342.

Ofori-Boateng C, Oduro W, Hillers A, Norris K, Oppong SK, Adum GB, Rodel MO. 2013. Differences in the effects of selective logging on amphibian assemblages in three west african forest types. Biotropica **45**:94–101.

Parris KM, Lindenmayer DB. 2004. Evidence that creation of a Pinus radiata plantation in south-eastern Australia has reduced habitat for frogs. Acta Oecologica **25**:93–101.

Pawar SS, Rawat GS, Choudhury BC. 2004. Recovery of frog and lizard communities following primary habitat alteration in Mizoram, Northeast India. BMC Ecology **4**:1–18.

Pineda E, Halffter G. 2004. Species diversity and habitat fragmentation: Frogs in a tropical montane landscape in Mexico. Biological Conservation **117**:499–508.

Pineda E, Moreno C, Escobar F, Halffter G. 2005. Frog, bat, and dung beetle diversity in the cloud forest and coffee agroecosystems of Veracruz, Mexico. Conservation Biology **19**:400–410.

Popescu VD, Patrick DA, Hunter ML, Calhoun AJK. 2012. The role of forest harvesting and subsequent vegetative regrowth in determining patterns of amphibian habitat use. Forest Ecology and Management **270**:163–174.

Rathod S, Rathod P. 2013. Amphibian communities in three different coffee plantation regimes in the Western Ghats , India. Journal of Threatened Taxa **5**:4404–4413.

Ribeiro-Júnior M, Gardner T, Ávila-Pires T. 2008. Evaluating the Effectiveness of Herpetofaunal Sampling Techniques across a Gradient of Habitat Change in a Tropical Forest Landscape. Journal of Herpetology **42**:733–749.

Russel K, Hanlin H, Wingley T, Guynn D. 2016. Responses of isolated wetland herpetofauna to upland forest management. The Journal of Wildlife Management **66**:603–617.

Santos Barrera G, Pacheco J, Mendoza Quijano F, Bolaños F, Cháves G, C. Daily G, R. Eirlich P, Ceballos G. 2008. Diversity, natural history and conservation of amphibians and reptiles from the San Vito Region, southwestern Costa Rica. Revista de Biología Tropical **56**:24.

Santos X, Poquet JM. 2010. Ecological succession and habitat attributes affect the postfire response of a Mediterranean reptile community. European Journal of Wildlife Research **56**:895–905.

Seshadri KS. 2014. Effects of historical selective logging on anuran communities in a wet evergreen forest, south India. Biotropica **46**:615–623.

Steen DA, Rall McGee AE, Hermann SM, Stiles JA, Stiles SH, Guyer C. 2010. Effects of forest management on amphibians and reptiles: Generalist species obscure trends among native forest associates. Open Environmental Sciences **4**:24–30.

Sung Y, Karraker NE, Hau BCH. 2012. Forest Ecology and Management Terrestrial herpetofaunal assemblages in secondary forests and exotic Lophostemon confertus plantations in South China. Forest Ecology and Management **270**:71–77.

Sutton WB, Wang Y, Schweitzer CJ. 2013. Amphibian and reptile responses to thinning and prescribed burning in mixed pine-hardwood forests of northwestern Alabama, USA. Forest Ecology and Management **295**:213–227.

Theisinger O, Ratianarivo MC. 2015. Patterns of reptile diversity loss in response to degradation in the spiny forest of Southern Madagascar. Herpetological Conservation and Biology **10**:273–283.

Trimble MJ, van Aarde RJ. 2014. Amphibian and reptile communities and functional groups over a land-use gradient in a coastal tropical forest landscape of high richness and endemicity. Animal Conservation **17**:441–453.

Vallan D. 2002. Effects of anthropogenic environmental changes on amphibian diversity in the rain forests of eastern Madagascar. Journal of Tropical Ecology **18**.

Vallan D, Andreone F, Raherisoa VH, Dolch R. 2004. Does selective wood exploitation affect amphibian diversity? The case of An’Ala, a tropical rainforest in eastern Madagascar. Oryx **38**:410–417.

Vonesh JR. 2001. Patterns of richness and sbundance in a tropical African leaf-litter herpetofauna. Biotropica **33**:502–510.

Wanger TC, Iskandar DT, Motzke I, Brook BW, Sodhi NS, Clough Y, Tscharntke T. 2010. Effects of land-use change on community composition of tropical amphibians and reptiles in Sulawesi, Indonesia. Conservation Biology **24**:795–802.

Warren-Thomas E, Menton M, Axmacher J. 2013. Frog communities in fire-disturbed forests of the Peruvian Amazon. Herpetological Bulletin **126**:12–24.
